# Engineering Hydroxyl Functionalization Enables Atomically Precise ZnO Nucleation on Defective Graphene

**DOI:** 10.1021/acsmaterialsau.5c00071

**Published:** 2025-08-13

**Authors:** Gaddiel Sandoval, Carlos Antonio Corona-Garcia, Jonathan Efrain Rodriguez Hueso, Mario Humberto Farías, Hugo Tiznado, Sergio Andres Aguila, H. A. Borbon-Nuñez, Jonathan Guerrero-Sanchez

**Affiliations:** † Facultad de Ingeniería, Arquitectura y Diseño, Universidad Autónoma de Baja California, 22860 Ensenada, Baja California , México; ‡ Centro de Nanociencias y Nanotecnología, Universidad Nacional Autónoma de México, 22800 Ensenada, Baja California, México; § Centro de Investigación Científica y de Educación Superior de Ensenada, Carretera Tijuana-Ensenada 3918, Apdo. Postal, 22860 Ensenada, Baja California, Mexico

**Keywords:** graphene, monovacancy, ZnO, hydroxyl functionalization, diethylzinc, atomic layer deposition, density functional theory

## Abstract

The synergy between graphene and ZnO in creating hybrid nanomaterials with novel properties of interest for the technological industry requires the development of processes and techniques that enable their precise production at the nanoscale. Therefore, understanding the atomic and molecular mechanisms that lead to their creation is imperative for controlling each involved step in their formation, enhancing their efficiency. This work sheds light on the first atomic layer half-cycle for the growth of ZnO on graphene with a hydroxyl-functionalized monovacancy. We performed quantum mechanical calculations, considering a trapping-mediated mechanism and diethylzinc (DEZ) as the precursor. The results suggest that neighboring hydroxyl groups facilitate DEZ adsorption and minimize the activation energy. This is linked to the role of hydroxyl groups in the formation of noncovalent interactions such as weak van der Waals and C–H···O and O–H···O hydrogen bonds, which stabilize the systems and facilitate the first partial reaction. By comparing the response in systems with one, two, and three hydroxyl groups, it was found that as these functional groups increased in quantity, the reactions were both thermodynamically and kinetically more favorable. Thus, it can be concluded that incorporating hydroxyl groups on graphene through pretreatment may considerably increase the initial growth rate of ZnO.

## Graphical abstract

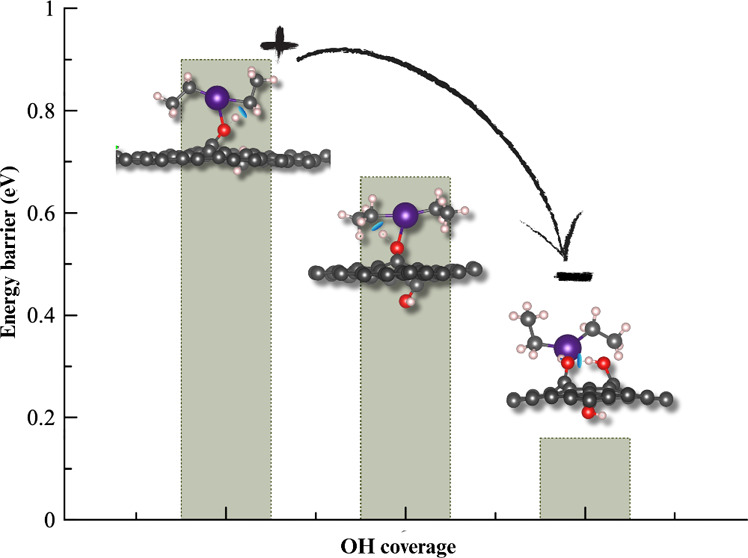

## 1 Introduction

The rapid technological development is pushing for an increasing demand for novel materials with unique and specific properties and applications. Two materials that have gained significant attention are graphene^1–5^ and zinc oxide (ZnO).^6–10^ Both have become relevant due to their extraordinary properties and wide range of potential applications. ZnO has gained special attention in the semiconductor industry due to its intrinsic bandgap (3.37 eV), null toxicity, high chemical stability, high isoelectric point, and electrochemical activity, making this material one of the most versatile transition metal-based oxides.^11–17^ ZnO has a wide range of applications such as light-emitting diodes, photodiodes, thin film transistors, and photovoltaic cells,^18–23,23^ just to mention a few.

On the other hand, graphene has excellent mechanical, electronic, and chemical properties that are widely known, such as its large surface area, excellent electrical and thermal conductivity, high electron mobility at room temperature, and chemical stability, among others.^24^ With the unique properties of graphene, the applications are vast, including flexible transistors,^25^ desalination membranes,^26^ optical sensors,^27^ and biosensors for detecting biomarkers.^28,29^ The main drawback of graphene is its lack of an intrinsic bandgap, which makes its application in micro and nanoelectronics applications^30^ difficult. A possible solution to balance this situation is to use graphene heterostructures with zinc-based materials.^16,31–35^ These heterostructures have opened up the possibility for new applications due to their intrinsic bandgap, high charge carrier mobility resulting from electronic interactions, improved catalytic and sensing activity, and enhanced electrical conductivity and photoelectric properties compared to their pristine counterparts.^16,36–38^

The graphene/ZnO-based heterostructures have been proposed for different applications, such as photocatalysts, pollutant molecule sensors, and transparent conductive thin films^34,38–43^ Experimentally, the fabrication of graphene/ZnO heterostructures has been reported using different techniques, such as electrodeposition,^40^ sol–gel,^39,43^ chemical vapor deposition (CVD),^16,44,45^ solvothermal,^38^ and oxygen plasma treatment method.^46^ However, a unique or uniform phase cannot be achieved with most of the mentioned methods.^47^ Moreover, using these fabrication techniques can introduce disorder in the atomic configuration of graphene, inducing adverse effects on its electronic, thermal, and magnetic properties.^31^ On the other hand, ALD is one of the emerging techniques for growing thin films, including semiconductor materials, due to the precise control over thickness and chemical composition, resulting in homogeneous growth materials.^31^ In ALD, surfaces play a central role; the chemical reactivity and inertia of the substrate surfaces are the nucleus of its functionality, making it necessary to understand the atomic-level mechanism involved.

Metal oxide deposition on graphene by ALD is challenging due to the lack of dangling bonds with unpaired electrons on its surface.^48^ Similarly, ALD of ZnO on pristine graphene has been reported as ineffective.^49^ In 2008, Wang et al. demonstrated that ALD of metal oxides does not result in direct growth on pristine graphene. This is due to the inert nature of the surface, arising from the delocalized π-bonds, which creates a stable system that limits covalent addition^48,50^ and, therefore, presents a low density of active sites for precursor adsorption. Consequently, ALD on pristine graphene usually leads to the production of films with low homogeneity or even the absence of film growth.^51^ In practice, modifying graphene surfaces by introducing defects in their structure is necessary, generating disorder that promotes the desired interaction with other molecules or atoms. Even current methods for synthesizing graphene do not guarantee a perfect surface, and the graphene produced contains vacancies, edges, curvatures, and chemical impurities.^50^ One option to promote the deposition on graphene-based materials is the introduction of functional groups in point defects, such as monovacancies, which provide anchoring sites for chemical precursors and enable uniform ZnO thin film growth. Although monovacancies have been experimentally shown to be stable^52,53^ and are associated with increased chemical reactivity and magnetic properties.^53,54^ Defects such as monovacancies exhibit a degree of mobility, and their migration is governed by activation barriers that depend on the defect nature and increase exponentially with temperature.^55^ This mobility can lead to the formation of more complex defects; for example, Yang et al.^56^ reported the formation of divacancies due to the coalescence of two monovacancies.

While divacancies are thermodynamically more stable than two isolated monovacancies,^57,58^ their influence on thin film growth is less favorable. Reconstructed divacancies often induce local curvature in the graphene lattice,^58–60^ leading to out-of-plane distortions that can compromise the structural properties and homogeneity,^61,62^ thereby inducing stress and deformations in the deposited layers that could lead to phase transition or phase combinations. Furthermore, divacancies have been shown to exhibit reduced chemical reactivity and to lose the magnetic properties associated with monovacancies,^54^ potentially altering the local electronic environment and decreasing precursor adsorption. Additionally, functionalizing is feasible since vacancies in the graphene basal plane are highly reactive.^50^ Introducing functional groups into the monovacancies is expected to have a double purpose: stabilizing defects by preventing their coalescence to complex defects^62,63^ and providing anchoring sites for molecules. These anchoring sites are particularly relevant for atomic layer deposition. In ALD processes, these sites are crucial since they act as interaction centers or nucleation sites for different phases in the material growth. For example, in the growth of ZnO by ALD, the substrate surface must contain oxygen species that facilitate the initial nucleation stages. Therefore, graphene oxide is commonly used as the substrate. The disadvantage in the use of graphene oxide is that it does not have a homogeneous composition and is characterized by presenting a significant number of structural defects and several functional groups, such as hydroxyl, epoxy, carboxyl, carbonyl, phenol, lactone, and quinone, among others.^50^ According to the structural model of graphene oxide proposed by Lerf and Klinowski, hydroxyl and epoxy groups are randomly distributed throughout the structure, whereas carboxyl and carbonyl groups are primarily located at the edges.^50,55^ Yu et al.^64^ suggested that graphene oxide contains many reactive hydroxyl groups within its structure, generating particular interest in investigating the functionalization of the monovacancies in graphene and graphene oxide (GO) to study the effect in the nucleation process. The selective growth of metal oxide onto a substrate is a process that can be achieved through the controlled introduction of activated sites. Mentel et al., activate predefined surface areas by functionalizing graphene using laser oxidation, to achieve excellent ALD selectivity.^65^ Effort has been put into the experimental study of graphene/ZnO heterostructures, but theoretical research of these heterostructures is necessary to understand the reaction mechanism to enhance the growth of homogeneous structures without affecting their properties. In this sense, Weckman and Laasonen have studied the adsorption mechanism of Diethylzinc (DEZ) on an ideal ZnO surface. Using first-principles calculations, they modeled the interactions of the ZnO surface with a DEZ molecule through functionalization with hydroxyl groups.^66^ Similarly, Guerrero-Sánchez et al.^67^ modeled the interaction of a hydroxylated carbon nanotube surface with a DEZ molecule, establishing the processes leading to its adsorption. Although the reported results provide significant insights into understanding the ZnO growth, the effects of the degree of functionalization during the initial nucleation phase have not yet been described.

Furthermore, the role of hydroxyl groups and monovacancies during the first ALD cycle remains unclear, particularly whether they merely serve as anchoring sites for the DEZ precursor or actively participate in the nucleation mechanism. For this reason, the present study focuses specifically on the first ALD half-cycle, the interaction between DEZ and functionalized graphene oxide with monovacancies to determine how these surface features influence the initial stages of ZnO growth. By evaluating various functionalization models and identifying the most stable configuration, we show that hydroxyl groups and vacancies not only enhance DEZ adsorption but also promote Zn incorporation, which is essential for subsequent oxygen uptake from water. These atomistic insights reveal that functionalized graphene oxide provides a reactive surface that facilitates ZnO nucleation, consistent with our experimental observations of crystalline ZnO growth. This union between theory and experiment underscores the pivotal role of surface chemistry in enabling uniform ZnO deposition on graphene-based materials. The evidence generated here provides deeper insights into the mechanisms involved in the formation of ZnO on graphene and other carbon allotropes.

## 2 Methods

### 2.1 Computational Method

Using spin-unrestricted quantum mechanical calculations, we investigated the influence of the hydroxyl groups on the interaction and stability between graphene and DEZ. The calculations were performed within the periodic Density Functional Theory (DFT) approximation as implemented in the PWscf code of the Quantum ESPRESSO package.^68^ The exchange-correlation energies were treated within the Generalized Gradient Approximation (GGA) in the Perdew–Burke–Ernzerhof parametrization (PBE).^69^ To describe accurately the long-range interactions, we consider van der Waals interactions as described in the Grimme-D_2_ method.^70^ The electron–ion interactions were modeled using the Vanderbilt Ultrasoft pseudopotentials (USP) with an optimized kinetic energy cutoff for the plane-wave basis set equal to 35 and 280 Ry for the charge density. The convergence in the relaxation process is reached by measuring the difference in energy between successive minimization steps; the energy difference is less than 1 × 10^–4^ Ry. A *k*-point grid of 2 × 2 × 1 was used for sampling the reciprocal space by following the Monkhorst–Pack scheme.^71^ The supercell method was employed to model the isolated molecule and functionalized graphene surface. The graphene surfaces were modeled using a 5 × 5 periodicity with a monovacancy at the center. A vacuum space larger than 17 Å was set in the normal direction to avoid interactions between replicas.

#### 2.1.1 Surface Formation Energy

As we are interested in investigating the thermodynamic stability of the monovacancy graphene interacting with different numbers of hydroxyl groups, it is necessary to determine the most stable structure after the adsorption of the hydroxyl groups in different sites near the monovacancy. With this in mind, we must employ the Surface Formation Energy (SFE) formalism, which is independent of the total number of atoms of each system (monovacancy graphene and hydroxyl groups) and only depends on the species’ chemical potential (μ). The SFE formalism is adapted from references:^10,72–74^

$$\mathrm{SFE}=\frac{E_{\mathrm{MG}-\mathrm{HG}}-E_{\mathrm{MG}}-\sum_{i}n_{i}\mu_{i}}{N}$$ 1

where *E*_MG–HG_ is the total energy of the monovacancy graphene interacting with the hydroxyl groups, *E*_MG_ is the total energy of the pristine monovacancy graphene, *n*_*i*_ and μ_*i*_ are the number of atoms and the chemical potential of the most stable phases of the *i*-th species that is not present in the reference system (*E*_MG_), and *N* is the total number of atoms forming the system. The chemical potentials are calculated from the following equation:

$$\mu =\frac{\partial E}{\partial N}$$ 2

where ∂*E* is the ground state system’s total energy and ∂*N* is the number of atoms of the system.^75^

#### 2.1.2 Nudged Elastic Band Method

The minimum energy path (MEP) for the studied reaction was determined using the Nudged Elastic Band (NEB) method^76^ as implemented in the Quantum ESPRESSO package. In the NEB calculations, we employed seven images between the optimized initial and final states, resulting in a total of 9 points along the path (including starting and ending points). Those images were connected through springs to prevent them from reaching the same local minima. The initial path was generated via linear interpolation. NEB was carried out using the Climbing Image (CI-NEB) method^77^ to describe the transition state accurately. We used a spring constant of 0.1 eV/Å^2^, and the force convergence threshold for each image was set to 0.05 eV/Å. All calculations were performed with an energy cutoff of 50 Ry. Both the initial and final structures were fully optimized prior to the NEB simulations.

### 2.2 Experimental Methods

Commercial powder of graphene nanoplatelets (Sigma-Aldrich, 900412) was used as a substrate for ZnO growth. The graphene nanoplatelets were dispersed by ultrasonication with a Cole Parmer ultrasonic probe operating at 750 W and 20 kHz to increase the surface area. Further, those were functionalized with hydroxyl groups through a photoinduction process by UV exposure in the presence of H_2_O_2_.

A thin ZnO film was grown on hydroxylated graphene using the atomic layer deposition (ALD) technique at 150 °C, in a powder reactor coupled to a Beneq TFS-200 system. The graphene powder (∼10 mg) was placed into the reaction chamber inside a metal capsule as sample-holder (2 cm^3^ powder capacity and 15 μm inlet/outlet pore size). Diethylzinc (DEZ from Strem Chemicals) and deionized water (H_2_O) were used as the zinc source and oxidizing agent, respectively. High-purity nitrogen at a flow rate of 200 sccm served as the carrier and purge gas.

Three deposits were performed to assess ZnO growth on graphene, consisting of 119, 60, and 30 cycles, each using 10 mg of graphene. Each ALD half-cycle included two precursor pulses, with a waiting time of 750 ms between pulses. The pulse durations were 75 ms for DEZ and 100 ms for water, while the purge time for both reactions was 7 s. The resulting coatings were chemically characterized using photoelectron spectroscopy on a SPECS system equipped with a hemispherical electron analyzer model PHOIBOS 150 WAL and a monochromatic X-ray source, of Al K_α_ (1486.6 eV) at 200 W. High-resolution spectra were taken at a pass energy of 20 eV. Functional groups were confirmed by FTIR spectroscopy recorded on a Bruker Tensor 27 Spectrometer in the 400–4000 cm^–1^ range.

## 3 Results and Discussion

### 3.1 Structural Properties

We first present the structural parameters of the graphene-optimized structure. Graphene crystallizes in a hexagonal structure with the *P*6_3_/*mmc* space group, and lattice parameters *a* = *b* = 2.47 Å, bond length between adjacent atoms of 1.42 Å, and inner angles of 120°, which agrees with other theoretical and experimental results.^63,78,79^

Several studies have examined the effect of the vacancies on graphene monolayers. Xu et al.^55^ report that asymmetric monovacancies are more stable than symmetric ones, but the asymmetric monovacancies give different results. In some cases, they keep the planar structure of the graphene,^80^ but other reports show that the vacancies promote out-of-plane displacements.^81,82^ In this study, we focus on 5 × 5 graphene monolayers with asymmetric monovacancies.

To study the effect of the vacancy on the structural properties of graphene, we compare the pristine and the monovacancy monolayers (Figure S1). Removing the central carbon atom on the monolayer generates three dangling sp^2^ bonds on the nearest carbon atoms, resulting in an asymmetric monovacancy due to the displacement of the nearest carbon atoms looking for the minimum energy configuration. In the monolayer in Figure S1b, the two nearest atoms to the vacancy form a weak bond of ∼2.15 Å, while the length with the other carbon atoms increases to ∼2.6 Å when compared with the lengths of the pristine system (2.47 Å). This structural configuration is called the Jahn–Teller planar distortion. Our results agree with the experiment and other theoretical results, as shown in Table 1.

**Table 1:** Theoretical and Experimental Length between the Nearest Carbon Atoms to the Vacancy

this work (Å)	other DFT works (Å)	experimental (Å)
2.15	2.1 ± 0.1 ^82,83^	1.9 ^62^
	2.40 ^84^	

According to Jing et al.,^80^ the reconstruction process has an essential role in stabilizing the Young̀s modulus because the introduction of vacancies disrupts the integrity of the pristine graphene monolayer, and Young′s modulus decreases. When an atom is removed from its position in the graphene lattice, the structure relaxes to a lower energy state by adjusting the bonds near the vacancy.^62,80^ Even though the surface reconstructions increase Young′s modulus, the asymmetric monovacancy graphene exhibits a lower value of the modules when compared with the pristine one.^85^ Therefore, the reconstruction process involving defects, like the Jahn–Teller distortion, contributes to surface stabilization by minimizing the system′s total energy, stabilizing Young′s modulus.

### 3.2 Defect Formation Energy

We calculate the defect formation energy (DFE) of the monovacancy as follows:^84^

$$E_{f}^{v}=E_{\mathrm{mv}}-N^{-1}(N-1)E_{\mathrm{PG}}$$ 3

where *E*_f_^v^ is the monovacancy formation energy, *E*_mv_ is the energy of the graphene with the monovacancy, *E*_PG_ is the energy of the pristine graphene structure, and *N* is the number of atoms that constitute the graphene supercell. The calculated *E*_f_^v^ value is 7.89 eV, which is in good agreement with the experimental (7.0 ± 0.5 eV^86^) and theoretical values (7.50–7.98 eV^84,87,88^ ). Although the required energy to induce monovacancies is high, these defects can occur spontaneously during the growth and processing of graphene.^55^ The main mechanisms leading to the generation of these defects include the irradiation with energetic particles, like electrons or ions, and chemical treatments. For example, the monovacancy formation in graphene can be locally controlled by manipulating an electron beam at 80 kV,^62^ making it possible to create covalent functionalization sites intentionally.

### 3.3 Monovacancy Functionalization

In this work, we are interested in evaluating the effects on the stability of the hydroxyl groups when they interact with the monovacancy in the graphene monolayer. The active sites that interact with the hydroxyl groups are the three carbons near the monovacancy. For this study, three systems were proposed, considering one, two, and three hydroxyl groups. It is worth mentioning that it was necessary to passivate the carbon atoms with dangling bonds by adding hydrogen atoms. This was mandatory because hydroxyl groups tend to dissociate in the presence of vacancies, forming stable ether groups after overcoming a low energy barrier of 0.22 eV.^89,90^ The passivation of carbon atoms with hydrogen is feasible since, according to Robertson et al.,^63^ hydrogen atoms tend to easily adsorb on the dangling bonds of the asymmetric monovacancy.

For the system with only one hydroxyl group adsorbed on the monovacancy passivated with two hydrogen atoms, several configurations were studied by varying the initial positions of the hydroxyl group on both sides of the graphene monolayer. Three stable configurations were obtained: 1OH_1_, 1OH_2_, and 1OH_3_, see Figures 1 and S2. In all three configurations, introducing the hydroxyl group induced a structural distortion and displacement of the nearest carbon atoms to the monovacancy, as already reported in the literature.^89^ Additionally, the hydroxyl group stands out of the graphene plane, while its hydrogen atom is preferentially oriented toward the monovacancy. The weak bond depicted in Figure S1b is broken due to the saturation of the carbon dangling bonds. As reported by Palacios and Ynduráin,^91^ the saturation of the monovacancy avoids the formation of the Jahn–Teller distortion. In all configurations, the adsorption of hydrogen and hydroxyl groups locally modified the graphene structure near the monovacancy, potentially forming hydrogen bonds (C–H···O).

**Figure 1:**
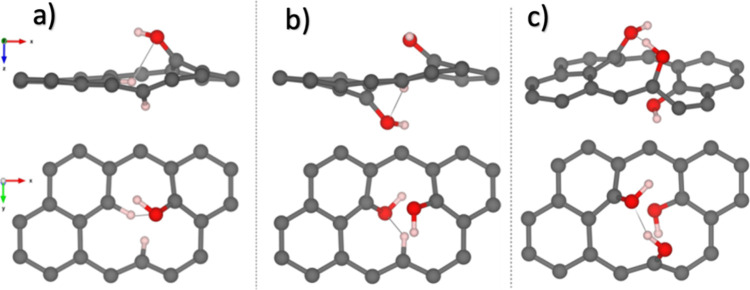
Side and top views of the most stable configurations for graphene with (a) one hydroxyl group (1OH_2_), (b) two hydroxyl groups (2OH_2_), and (c) three hydroxyl groups (3OH_2_). Gray, white, and red spheres represent carbon, hydrogen, and oxygen atoms.

Two stable configurations were found for two hydroxyl groups, the 2OH_1_ and 2OH_2_ (see Figures 1b and S2). In the 2OH_1_ configuration (Figure S2c), both hydroxyl groups are positioned on the same side of the monolayer, while the hydrogen atom that passivates the carbon dangling bond lies in the same plane. A potential hydrogen bond (O–H···O) is observed between the two hydroxyl groups. On the other hand, for the 2OH_2_ configuration (Figure 1b), both hydroxyl groups are on opposite sides of the graphene monolayer, and the hydrogen atom is slightly oriented toward the hydroxyl group below the monolayer. Similar to the 2OH_1_ configuration, a potential hydrogen bond is formed. Still, for the 2OH_2_ configuration, it is between the hydroxyl group and the hydrogen atom adsorbed at the monovacancy (C–H···O).

For the graphene monolayer functionalized with three hydroxyl groups, of the different initial configurations tested, only two structures were stable: the 3OH_1_ and 3OH_2_ (Figures 1c and S2). The 3OH_1_ configuration (Figure S2d) has all the hydroxyl groups on the same side of the monolayer. The stability of this structure is achieved through the formation of three possible hydrogen bonds (O–H···O) between the hydroxyl groups, with an O–O bond length of 2.47 Å and bond angles of 137.8 and −138.4°. For the 3OH_2_ configuration (Figure 1c), two hydroxyl groups are on the same side of the monolayer, while the third is on the opposite side. In this case, the stability is due to the possible formation of hydrogen bonds (O–H···O) between the two hydroxyl groups on the same side of the monolayer. The O–O bond distance is 2.40 Å, and the bond angle is 143.7°.

### 3.4 Thermodynamic Stability

We use the surface formation energy formalism to compute the stability of the monovacancy graphene when interacting with the hydroxyl groups. Since this formalism is independent of the number of atoms and only depends on the chemical potentials,^10,72–74^ it is possible to compare the different structures to determine the most stable.

#### 3.4.1 Surface Stability

The surface formation energy values are shown in Table 2. As noticed in Table 2, the SFE values are approximately −0.135 eV/Å^2^ (±0.015), which indicates that the saturation of the dangling bonds is highly favorable. This has been confirmed experimentally by Ziatdinov et al.^92^ They demonstrated that the saturation of monovacancies with hydrogen atoms is feasible. Additionally, they observed that the dangling bonds preferentially bond with a single hydrogen atom when the chemical potential is below −3.66 eV.

**Table 2:** Surface Formation Energy (SFE) Values of the Interaction of Monovacancy Graphene with One, Two, and Three Hydroxyl Groups

configuration	OH	H	H bonds	SFE (eV/Å^2^)
1OH_1_	1	2	0	–0.122
1OH_2_	1	2	1	–0.123
1OH_3_	1	2	1	–0.122
2OH_1_	2	1	1	–0.135
2OH_2_	2	1	1	–0.148
3OH_1_	3	0	3	–0.137
3OH_2_	3	0	1	–0.158

For the systems interacting with only one hydroxyl group, the SFE values are almost degenerate, with a difference of just 0.001 eV/Å^2^, which indicates that all configurations are equally probable. However, in the works by Paez et al.^93^ and Matsutsu et al.,^89^ all the tested configurations converged into a single structure similar to our 1OH_3_. In those works, they do not mention or clarify whether the monovacancy is symmetric or asymmetric, so they may not consider the potential effects of the Jahn–Teller distortion in their results.

In the systems with two hydroxyl groups, the SFE difference is just 0.013 eV/Å^2^, with the 2OH_2_ being the most stable. For the systems with three hydroxyl groups, the most stable configuration is the 3OH_2_, with an SFE difference of 0.021 eV/Å^2^ compared to the 3OH_1_ structure. As shown in Table 2, the SFE tends to decrease as the number of hydroxyl groups increases, making the 3OH_2_ configuration the most stable.

Something to note is that, according to SFE, the most stable structures of the systems with two and three hydroxyl groups are when two hydroxyl groups are in different planes of the monolayer, notice that 2HO_2_ is more stable than the 3OH_1_ even when the 3OH_1_ configuration falls into a spatial configuration that suggests the formation of three hydrogen bonds between the hydroxyl groups, the 3OH_2_ configuration with only one hydrogen bond, and the 2HO_2_ are more stable. This effect has also been observed in the adsorption of a pair of hydrogen atoms^94^ and in the adsorption of hydroxyl groups in pristine graphene and other kinds of defects, like the Stone-Wales.^90^

Based on the SFE results, the discussion will focus only on the most stable structures for one, two, and three hydroxyl groups from this point onward.

### 3.5 ZnO Nucleation

In atomic layer deposition of ZnO, the most common precursors used are DEZ and H_2_O.^51^ The reaction mechanism for the first ALD half-cycle to deposit this material begins with the adsorption of DEZ molecules onto the nucleation centers, where the hydroxyl groups are bonded to the surface (in our case, graphene).^93,95^ Once this interaction occurs, the reaction proceeds through a mediated capture mechanism in which the hydroxyl group acts as a hydrogen donor. This involves a ligand exchange with the DEZ molecule, forming monoethylzinc (MEZ),^66^ bonded to oxygen (O-MEZ), and the recombination of a departing C_2_H_5_ ligand with the hydrogen atom donated by the hydroxyl group, resulting in an ethane molecule (C_2_H_6_) as subproduct.^93,95^ Due to its electronegativity difference, the hydroxyl group can easily deprotonate in this process. Similarly, the DEZ molecule dissociates through the breaking of the Zn–C bonds. This has been confirmed by Kim et al.,^96^ who used the Wiberg bond index in the natural bond orbital (NBO), finding that the Zn–C bond is the weakest in the DEZ molecule. Additionally, due to the empty low-energy sp orbitals on the zinc atom, the metal–carbon covalent bond becomes highly sensitive to proton sources,^97^ so it can be dissociated in the presence of hydroxyl groups. As Ren^98^ suggested, the O-MEZ complex is formed through the interaction between the free pair from the oxygen, previously part of the hydroxyl group, and an empty hybridized sp orbital from the divalent zinc.

#### 3.5.1 Minimum Energy Pathways for the ZnO Nucleation

To analyze the influence of the hydroxyl groups on the reaction mechanism associated with the ZnO nucleation on graphene, the nudged elastic band method was employed to determine the minimum energy pathways for converting reactants into products. Different configurations for the initial (IS), transition (TS), and final states (FS) of the most stable structures were obtained with the SFE analysis. In the initial state, the adsorption of DEZ on the hydroxyl groups was considered. According to Thian et al.,^96^ ALD growth of ZnO on silicon using DEZ as a precursor can occur at sites terminated with hydrogen atoms and hydroxyl groups.^96^ However, due to the pronounced out-of-plane displacements of the hydroxyl groups compared to the hydrogen atoms on graphene (Figures 1 and S2), physisorption on hydroxyl groups is preferred.

The reaction mechanisms for the 1OH and 2OH are shown in Figure S3 from the Supporting Information. Both systems follow a similar pathway because the hydroxyl group in the 2OH system is located on opposite planes of the graphene monolayer, and the DEZ molecule interacts only with one hydroxyl group, similar to the 1OH system. In both systems, the DEZ molecule adsorbs onto the hydroxyl group bonded to the graphene at the IS. To reach TS, the reaction needs additional energy. The energy barrier is 0.90 and 0.67 eV for the 1OH and 2OH systems, respectively (see minimum energy pathways in Figure S3a,b). Similar results were found in the literature for a hydroxylated ZnO^100^ surface,^66^ and a hydroxylated silicon surface.^99^ Our results, in combination with the literature, evidence that the activation energy is determined by the interaction between the hydroxyl species and the DEZ rather than by the substrate surface. In the TS of both systems, the partial extraction of the hydrogen atom from the hydroxyl group and the dissociation of the Zn–C bond from the DEZ are observed. A partially formed Zn–O bond with a bond distance of 2.22 Å appears. In the FS, the O-MEZ complex is formed, and the hydrogen atom is completely extracted from the hydroxyl group, interacting with the dissociated ethyl group from the DEZ molecule, forming an ethane molecule. Both reactions are exothermic by 0.79 and 1.01 eV for the 1OH and 2OH structures, respectively, indicating that it is thermodynamically favorable for both systems.

The minimum energy pathways for the ZnO nucleation onto graphene with three hydroxyl groups are shown in Figure 2. According to the SFE, the introduction of a third hydroxyl group reduces the energy barrier to just 0.16 eV, being the lowest of the three systems under study. Similar to the 1OH and 2OH, in the IS1 for the 3OH structure, the DEZ molecule adsorbs onto one of the hydroxyl groups in the same graphene plane. Unlike the 1OH and 2OH systems, in the TS1 of the 3OH system, the O–H bond from the hydroxyl group does not dissociate but generates a reconfiguration of the DEZ molecule. The C–C–Zn angles were modified to 105.72° for the ligand far away from the interaction (left part of the DEZ molecule) and 122.28° for the ligand that forms the O-MEZ complex. For comparison purposes, the relaxed DEZ molecule from the Supporting Information is shown in Figure S4. Another difference with respect to 1OH and 2OH systems is that for the 3OH structure, neither the premature Zn–O bond nor the Zn–C breaking bond from the DEZ are observed. However, in the FS1, the ethane molecule and the O-MEZ complex formation is achieved with an exothermic reaction of 1.22 eV, which is thermodynamically more favorable than the 1OH and 2OH systems. As shown in Table 3, variations in bond distances throughout the pathways confirm the breaking of O–H and Zn–C bonds, except for the 3OH system, and the formation of ZnO at the final state for all structures. The Zn–O bond length from the FS state of the three systems is close enough to the reported values of 1.83 Å^93^ and 1.82 Å,^66^ see Table 3 and Figure S5 in the Supporting Information for structural details for he IS and FS states.

**Figure 2:**
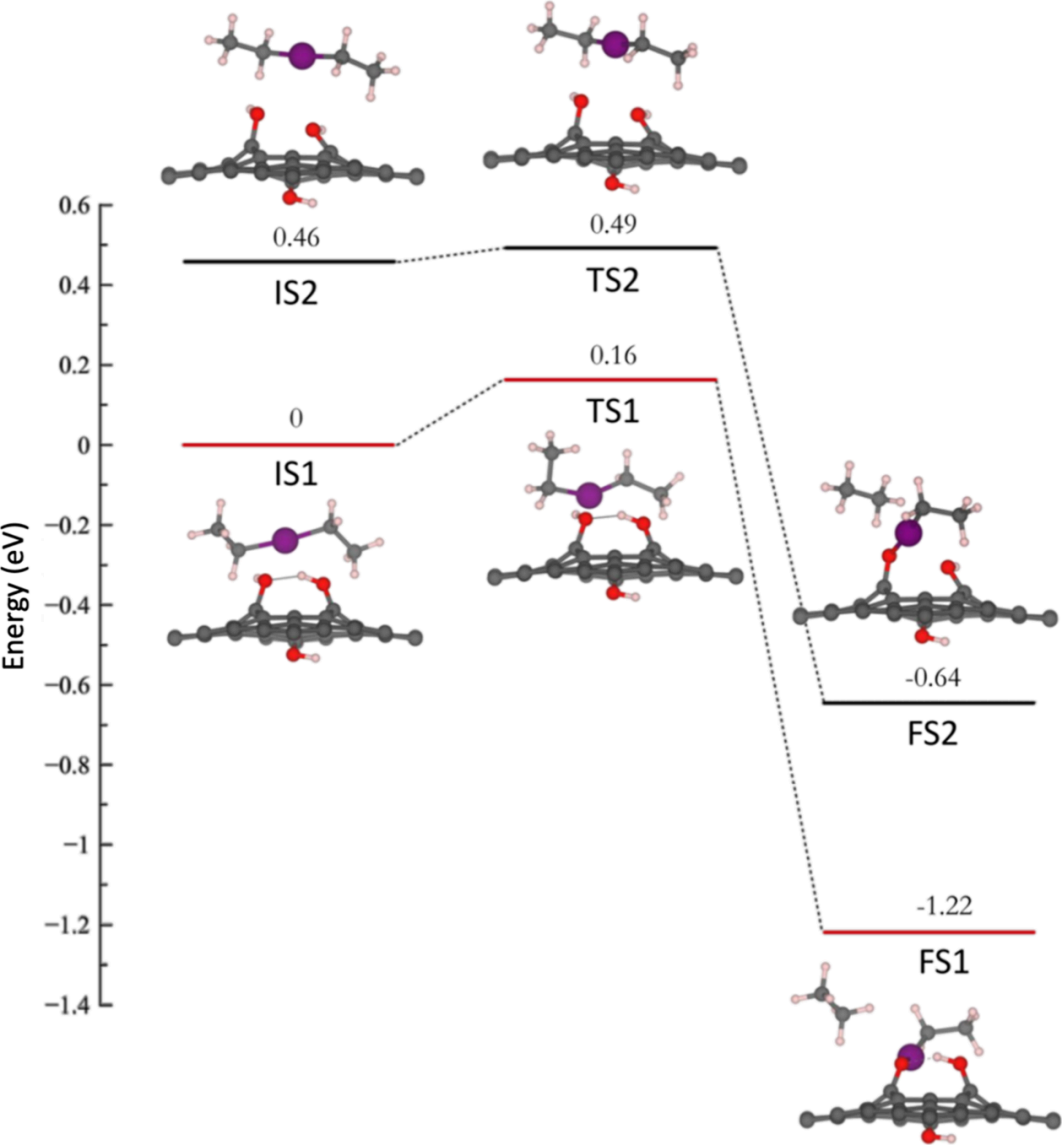
Minimum energy pathway of the first partial reaction of monovacancy graphene with three hydroxyl groups. IS1, TS1, and FS1 states correspond to the system where the hydrogen bond is considered, and IS2, TS2, and FS2 correspond to the system where the hydrogen bond was omitted.

**Table 3:** Interaction Distances (Å) between the Main Atoms in the Partial Reaction of DEZ for the Most Stable Structures

system	initial state				transition state				final state
	Zn–O	Zn–H	Zn–C	O–H	Zn–O	Zn–H	Zn–C	O–H	Zn–O
1OH	3.22	3.12	1.96	0.98	2.22	1.79	2.21	1.27	1.84
2OH	2.72	2.77	1.97	0.99	2.18	1.87	2.15	1.25	1.89
3OH	3.28	2.61	1.97	0.99	3.44	2.58	1.98	1.00	1.97

To demonstrate the important role of the hydrogen bonds in the stabilization of the 3OH system and the reduction of the SFE and the activation energy, an alternative system was analyzed in which the hydrogen bond was omitted. Figure 2 shows the pathways for the proposed reaction with labels IS2, TS2, and FS2. The reaction mechanism is similar to the original system. Still, the energy barrier increases to 0.49 eV, three times bigger than the one with hydrogen bonds. The reaction is still exothermic with a gain in energy of 0.64 eV when reaching the FS2 state. With this proof of concept, we have demonstrated that hydrogen bonds play a critical role in stabilizing the systems and controlling the energy barriers for achieving an engineered first-half ALD reaction.

### 3.6 Noncovalent Interactions

Previously, it was mentioned the formation of weak bonds between carbon atoms near the monovacancy in the graphene monolayer. Also, the existence of hydrogen bonds when hydroxyl groups interact with the monovacancies in the graphene monolayer. We used the noncovalent interactions analysis (NCI) (see Theoretical section from the Supplementary Material)^100–103^ to confirm the weak and hydrogen bonds. The isosurfaces of the NCI are represented in an RGB scheme, where it is possible to define three kinds of interactions: nonbonding interactions or steric effects (red), weak interactions or van der Waals (vdw) interactions (green), and bonding interactions (blue).

#### 3.6.1 Noncovalent Interactions of Graphene with Monovacancy

First, we present the NCI isosurfaces for the graphene with a monovacancy. As shown in Figure 3a, noncovalent interactions are observed exclusively around the monovacancy region and at the center of each carbon hexagon. The orange-colored centers represent regions of repulsion where electronic clouds overlap. In the monovacancy region, the lobes of the three sp^2^ orbitals from carbon atoms with dangling bonds point toward the vacancy center, creating a region of strong interactions between the two atoms and forming the reconstructed weak bond from Figure 1. Similar results as the reported by Nanda et al.^84^ Additionally, a weak attractive interaction is present between the atom with the dangling bond and each of the two atoms forming the reconstructed bond, separated by approximately ∼2.6 Å.

**Figure 3:**
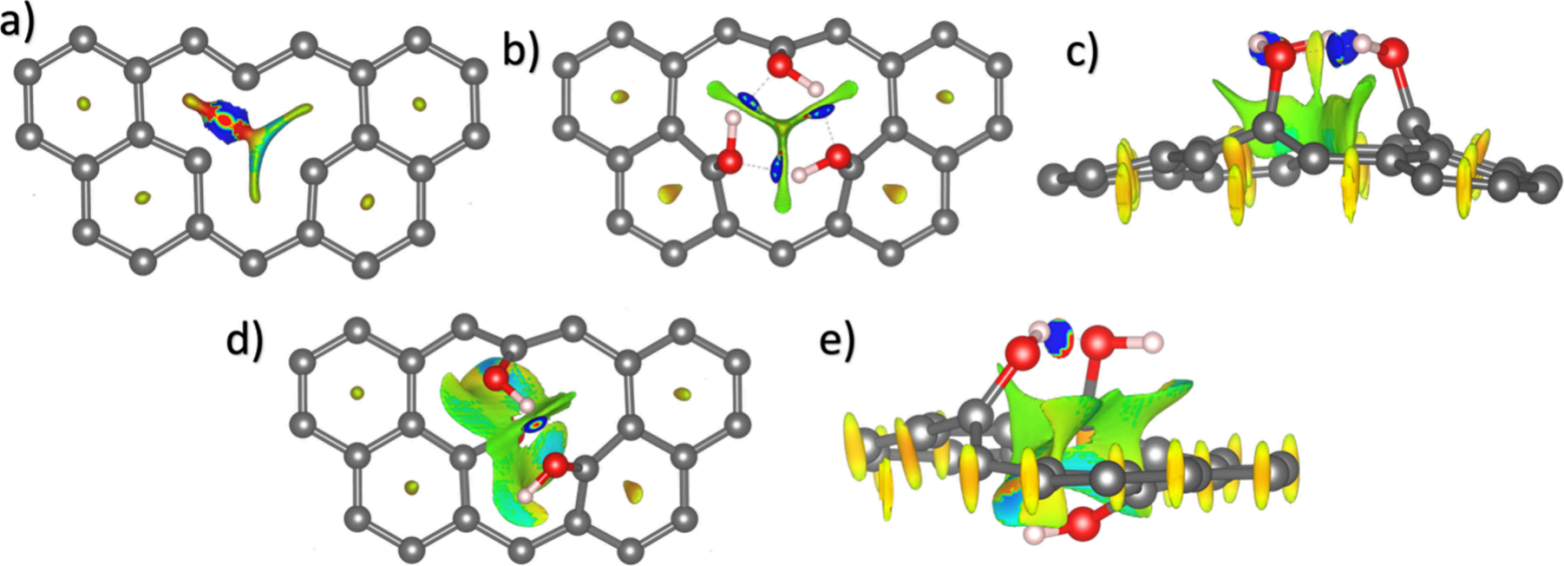
Isosurface of the NCI of the 5 × 5 graphene monolayer with an asymmetric monovacancy with Jahn–Teller distortion for the (a) top view of the pristine system, and with three hydroxyl groups for the (b) top and (c) side views of the 3OH_1_ structure, and (d) top and (e) side views 3OH_2_ structure.

Additionally, at the edges of this attractive region, repulsive interactions arise due to the influence of the nearest carbon atoms that form the monovacancy. A repulsion center can also be identified between the attractive region and the reconstructed bond, where the paired electrons of the reconstructed bond repel the unpaired electron of the atom with the dangling bond.^82^ This repulsion, combined with the edge repulsion of the attractive region, reduces the bond length between the dangling carbon atom and its nearest neighbor carbon atom to 1.37 Å, a result that is consistent with the experimental report of Robertson et al.^62^ In contrast with the report by El-Barbary et al.,^82^ they state that the repulsion between the paired electrons of the reconstructed bond and the unpaired carbon electron does not induce the displacement of this atom out of the graphene plane. Instead, this is mainly due to the attractive interactions with the nearest carbon atoms of the monovacancy, which keep the dangling atom in the monolayer plane.

#### 3.6.2 Noncovalent Interactions of Functionalized Monovacancy Graphene

In this section, we show the NCI isosurfaces only for the 3OH configurations of the monovacancy graphene. According to the SFE results, the most stable structure is the 3OH_2_ (Figure 1c), but just for comparison, we show also the 3OH_1_ structure (Figure S2d). Figure 3b–e shows the NCI isosurface for the 3OH_1_ and 3OH_2_ systems. Similar to the monovacancy graphene, the orange spheres in the center of the graphene hexagons correspond to repulsions. In contrast with the monovacancy graphene, the van der Waals interactions (green regions) between the carbon atoms near the monovacancy are better defined when the hydroxyl groups interact with the monovacancy.

Additionally, the NCI confirms the formation of the hydrogen bond (O–H···O). In the 3OH_1_ system, blue lobes emerge between the three hydroxyl groups, while for the 3OH_2_ system, the hydrogen bond is observed between the two hydroxyl groups on top of the monolayer. In the 2OH_2_ configuration (Figure S6), it is also possible to observe the attractive interactions between the hydrogen and oxygen atoms of the hydroxyl group and the carbon atoms at the edge of the monovacancy that are below the monolayer. In the side view of the NCI (Figure 3e) it is possible to notice that the area where vdW interactions happened is bigger than that the observed in the 3OH1 system (Figure 3c). This indicates that vdW interactions have an essential role in stabilizing the system. Similar results were found for the 1OH_2_ and 2OH_2_ systems.

#### 3.6.3 Noncovalent Interactions of the ZnO Nucleation

Figure 4 shows the NCI isosurfaces of the first half cycle of the ZnO nucleation on monovacancy graphene functionalized with three hydroxyl groups. The NCI for the systems with one and two hydroxyl groups is shown in Figures S6 and S7 from the Supporting Information.

**Figure 4:**
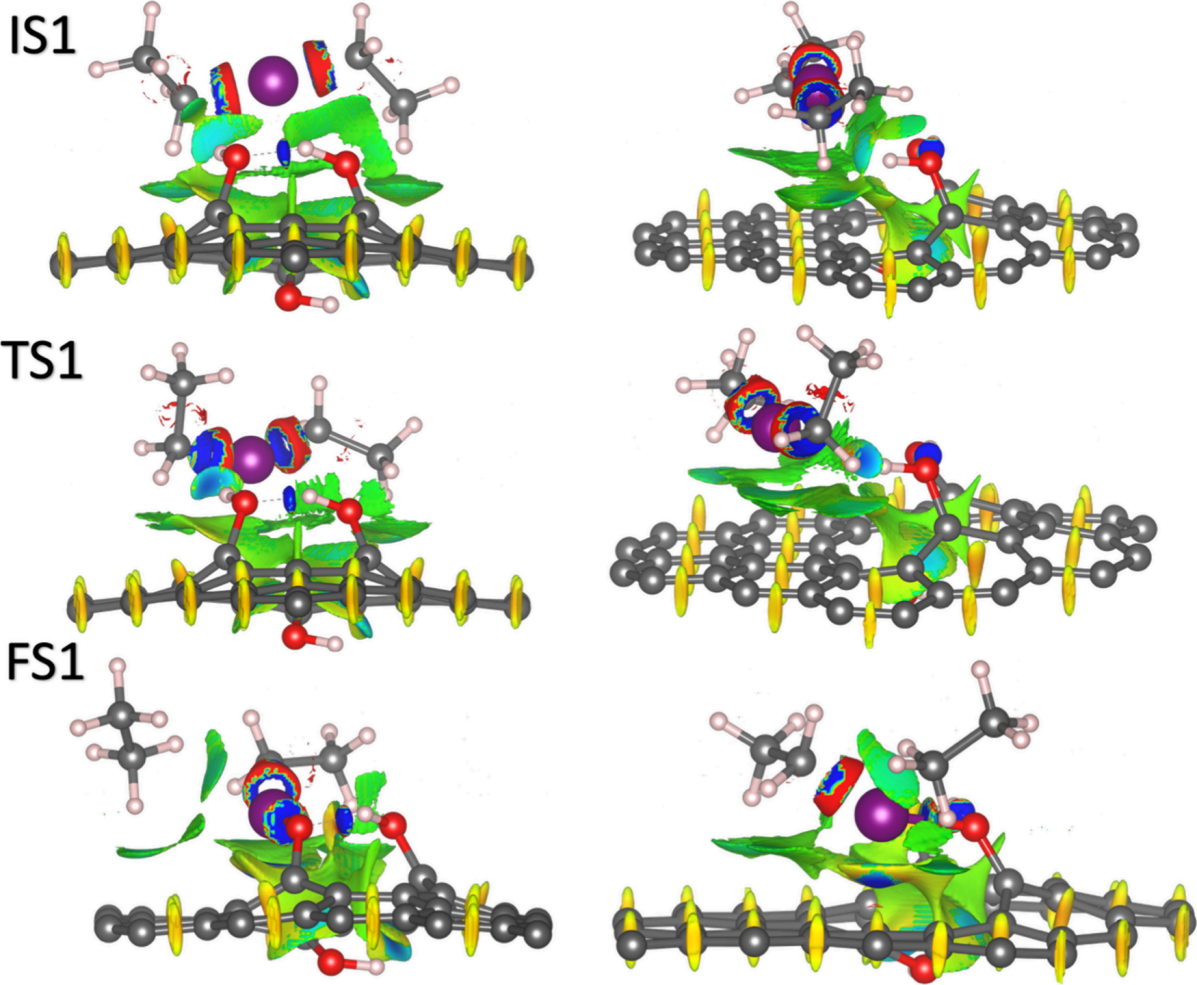
NCI isosurfaces of the first ALD half cycle for the ZnO deposition on monovacancy graphene functionalized with three hydroxyl groups.

For all the systems, the initial states of the physisorption are through van der Waals interactions between the DEZ molecule, the graphene surface, and the hydroxyl groups. The van der Waals interactions magnify as the number of hydroxyl groups increases. The van der Waals interactions mainly occur between the hydroxyl groups and the carbon atoms at the edges of the monovacancies. Additionally, the hydroxyl groups in all three systems generate electrostatic interactions and strong attraction forces. In the 3OH system, an O–H···O hydrogen bond forms between the hydroxyl groups in the same plane of the monolayer. van der Waals interactions are observed between the DEZ molecule and the hydroxyl group involved in the hydrogen bond.

The TS1 of the interactions between the DEZ molecule and the hydroxyl group behaves similarly in the 1OH and 2OH systems. At this state, the hydroxyl group dissociates completely, generating a strong interaction between the Zn and O atoms, demonstrating the formation of the Zn–O bond. For the 2OH system, the presence of a second hydroxyl group spread out the noncovalent interaction region in the vicinity of the monovacancy area, helping in the formation of a double hydrogen bond between the two hydroxyl groups and the hydrogen atom in the monovacancy, contributing to a stronger physisorption of the DEZ molecule, and reducing the energetic barrier, when compared with the 1OH system. In the 3OH system, as we discussed earlier, the TS is characterized by the reconfiguration and rotation of the DEZ molecule instead of the O–H bond dissociation. This reduces van der Waals interactions with the hydroxyl groups, forming the hydrogen bond and allowing a strong interaction region (blue lobes) between the hydrogen atom of the hydroxyl group and the out-of-plane carbon atom at the middle of the monolayer, see Figure 4.

Additionally, this increases the interaction with the monolayer through weak attractions and van der Waals interactions. These interactions orient the DEZ molecule in a way that facilitates ligand exchange, resulting in a lower energy barrier. Since for the 3OH system, the DEZ molecule does not dissociate, and the exchanged hydrogen atom remains bonded, this system is more stable than the 1OH and 2OH structures.

In the FS of all three cases, the MEZ is physisorbed through van der Waals interactions between the ligand hydrogens and the graphene surface carbons (see Figures 4, S6, and S7). Similar interactions are observed between the ethane molecule and the graphene. In the 2OH and 3OH systems, the Zn atom is physisorbed onto two carbon atoms at the edge of the monovacancy through attractive interactions. But for the 3OH structure, a weak attraction region is observed between the Zn atom and the O atom of the hydroxyl group involved in the hydrogen bond. The difference in the FS Zn–O bonds of the 3OH system when compared to 1OH and 2OH systems is attributed to this weak interaction.

The energy barrier reduction in the 3OH system is mainly due to the additional attractive interaction between the free electron pair of the O atom from the second hydroxyl group and an empty hybridized sp orbital of the divalent zinc ion. Our results align with those reported by Ren^98^ on silicon surfaces, where the reactions involving two nearby hydroxyl groups are thermodynamically and kinetically favorable.

The surface curvature may result in interactions with the carbon atoms at the edges of the monovacancies. According to El-Barbary et al.,^82^ this is due to the weakening of the π bonds caused by the reduction of the p_*z*_ atomic orbital overlap when the distance and angles between carbons change from their ideal structure. This promotes the rotation and rehybridization of their orbitals,^104^ increasing their reactivity.^50^

In all three systems, the DEZ molecule’s physisorption main interactions are the attractive and van der Waals interactions, which are presented in the first cycle of the ZnO nucleation. These interactions are crucial for understanding chemical reactions on material surfaces. Based on our results, hydroxylation of substrates for ZnO nucleation in the first ALD half-cycle is favorable, as reactions with nearby hydroxyl groups significantly reduce the energy barrier.

### 3.7 ZnO Growth onto Graphene

In this work, we also grow ZnO onto commercial graphene oxide by ALD at 150 °C, to confirm the homogeneous deposition. *FTIR spectroscopy was employed to identify the main functional groups present in the graphene oxide and in the graphene/ZnO heterostructure* (Figure 5a). The functionalized graphene FTIR spectra show a broad peak at 3428 cm^–1^ attributed to the presence of hydroxyl groups,^105^ while the peaks at 2922 and 2853 cm^–1^ correspond to the C–H stretch bonding in sp^3^ hybridization.^106^ Also, the peak at 1734 cm^–1^ corresponds to the −C=O groups.^107^ The peaks at 1629 cm^–1^,^106,107^ 1384 cm^–1^,^105,108^ and 1114 cm^–1^ ^105^ are associated with the stretching mode of carbon atoms in sp^2^ hybridization within the graphene lattice,^106,107^ the stretching vibrations of the C–OH bond,^105,108^ and to the in-plane torsional vibrations of the C–H bonds,^105^ respectively. Prior to the functionalization process, graphene nanoplatelets exhibited peaks indicative of graphitic carbon, accompanied by a weak band attributed to hydroxyl groups. These results confirm the successful functionalization achieved through photoinduction. Regarding the heterostructure, a similar set of peaks is observed, similar to those in graphene oxide, with an additional peak at 424 cm^–1^, which is attributed to the Zn–O bond.^109^ This indicates that functionalized graphene was not modified during the ALD process; even the peak at 3428 cm^–1^ remains in the same ratio as the peak at 2922 cm^–1^. However, this is attributed to the presence of adsorbed water on the surface,^107^ due to the hydrophilic nature of GO and the exposure to water in the ALD process.^105^

**Figure 5:**
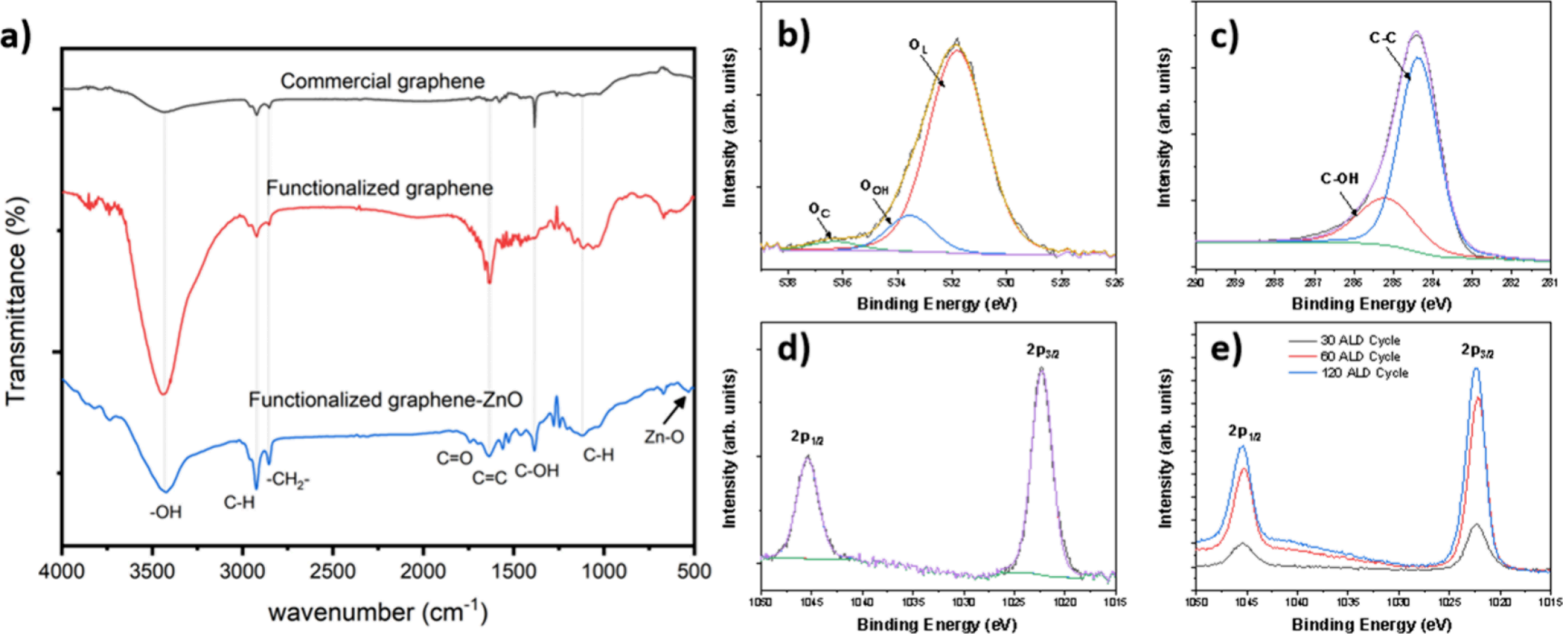
(a) FTIR spectra of GO (black line), functionalized GO (red line) and GO/ZnO heterostructure (blue line), and XPS high-resolution spectra of (b) O-1s, (c) C-1s, and (d) Zn-2p, and (e) a comparison of the Zn 2p signal, as a function of the number of ALD cycles.

The chemical composition of the graphene/ZnO heterostructure was confirmed using XPS characterization technique. Figure 5 shows the High-resolution XPS (HR-XPS) spectra for oxygen, carbon, and zinc, after 30 ALD cycles. Figure 5b shows the HR-XPS spectra of O-1s oxygen fitted by three nearly Gaussian components centered at 532, 533, and 536 eV, where the lower binding energy component is attributed to oxygen in hexagonal wurtzite structure of the metal oxide (O_L_), Zn–O bonds,^110,111^ and the higher binding energy peaks are associated with surface adsorbed species such as carbonates/bicarbonates and hydroxyl species, and to chemisorbed water or other oxygen species (O_C_), respectively.^112–114^ The C-1s high-resolution peak is observed in Figure 5c, fitted by two individual peaks, centered at 284.4 and 286 eV, indicating that two different chemical environments of carbon existed in the heterostructure. The lower binding energy component is assigned to the contributions of −C–C– bonds, while the peaks at higher binding energy are attributed to oxidized carbon species, in this case – COH.^115,116^ Figure 5d shows the Zn 2p HR-XPS spectra, where the presence of two peaks is associated with Zn 2p_3/2_ (1022 eV), Zn 2p_1/2_ (1045 eV).^115,117^ The binding energy difference between the two lines for Zn 2p is 23 eV, which shows that Zn atoms are in a 2^+^ oxidation state, according to the standard reference value for ZnO,^117,118^ indicating that Zn in the heterostructure existed in its oxidized state. This result confirms the presence of hydroxyl groups at the surface of functionalized graphene, as well as the presence of ZnO in the heterostructure.

To analyze the behavior of the chemical species present in the coated graphene as a function of ALD cycles, the HR-XPS spectra of carbon, oxygen, and zinc are shown in Figure S8. No significant changes in the chemical species are observed. After 60 cycles, the oxygen species associated with chemisorbed water or other oxygen species are absent in this sample, although they are present in low concentrations in others. For carbon species, a weak and broad band located at 288.8 eV, associated with O–C=O, is observed for 30 and 60 ALD cycles. However, this band is not observed when graphene is exposed to 120 cycles. This change may be attributed to the chemisorption of DEZ on various active sites of the graphene surface. As DEZ exposure to the surface increases, all the active sites, i.e., carbonyl groups, react to form a conformal coating on the surface. In the case of zinc species, there are no changes in chemical species or differences between the two spectral lines. However, a higher intensity of signal peaks is observed as the ALD cycles increase (Figure 5e).

## 4 Conclusions

We studied the functionalization of monovacancy graphene with hydroxyl groups and the initial growth of ZnO onto monovacancy graphene through first-principles calculations. Our results indicate that asymmetric planar monovacancies in graphene result from the attractive interaction between a carbon with a dangling bond and each of the two carbons that form the weak bond. When chemical species adsorb onto these defects, each dangling bond becomes saturated, breaking the weak bond. Introducing adsorbates into two-dimensional materials like graphene tends to minimize the system’s total energy by distributing them on both sides of the monolayer, thereby reducing structural deformation. For the adsorption of hydroxyl groups and hydrogen atoms on monovacancy graphene, the surface formation energy is at least −0.122 eV. The presence of nearby hydroxyl groups can facilitate DEZ adsorption and lower the activation barrier, making hydroxyl group incorporation highly favorable during the nucleation process in atomic layer deposition of ZnO on defective graphene. This suggests that hydroxyl groups play a key role in nucleation by promoting noncovalent interactions that stabilize the system and help zinc incorporation in the initial reaction. These interactions include van der Waals forces, attraction, and hydrogen bonds such as C–O···O and O–H···O. Experimental results confirm ZnO growth on graphene oxide using ALD, as shown by FTIR and XPS analysis. The FTIR spectrum indicates that graphene oxide remains unreduced during the ALD cycle at 150 °C, with main functional groups preserved, implying the functionalized surface remains intact throughout deposition. These observations support the theoretical prediction that hydroxyl groups and single vacancies are crucial in the early stages and stability of ZnO growth on carbon-based materials. Using graphene oxide as a model, we anticipate that other carbon-based materials with sp^2^ bonds and controlled defect structures, such as graphite and nanotubes, will exhibit similar results, broadening the range of substrates suitable for growing ZnO and other materials.

## Supplementary Material



## Notes

Top and side views of pristine and monovacancy graphene systems; top and side views of the less stable configurations of monovacancy graphene interacting with one, two, and three hydroxyl groups, for comparison purposes; minimum energy pathways of the first partial reaction for the less stable configurations of monovacancy graphene with one and two hydroxyl groups; bond lengths and angles of the DEZ molecule to allow comparison before and after interaction with the graphene system; side views of atomistic models of the DEZ partial reaction at the initial, transition, and final states for the most stable configurations; noncovalent interaction between the monovacancy graphene with one and two hydroxyl groups and the DEZ molecule through the reaction path; and brief discussion on the theoretical background of noncovalent interactions (PDF)
