# Supplementary material for: Engineering Hydroxyl Functionalization Enables Atomically Precise ZnO Nucleation on Defective Graphene
Source: ACS Mater Au. 2025 Aug 13;5(6):978–90. doi: 10.1021/acsmaterialsau.5c00071 (PMC12616442; doi:10.1021/acsmaterialsau.5c00071)
Supplement: Supplementary file 1 [file mg5c00071_si_001.pdf]

Supporting Information of the article:

Engineering Hydroxyl Functionalization Enables Atomically Precise ZnO Nucleation on Defective Graphene

Gaddiel Sandoval[1], Carlos Antonio Corona-Garcia[2], Jonathan Efrain Rodriguez Hueso[2,3], Mario Humberto Farías[2], Hugo Tiznado[2], Sergio Andres Aguila[2], H. A. Borbon-Nuñez[1*], Jonathan Guerrero-Sanchez[2*]
[1] Facultad de Ingeniería, Arquitectura y Diseño, Universidad Autónoma de Baja California, Ensenada, Baja California, México.
[2] Centro de Nanociencias y Nanotecnología, Universidad Nacional Autónoma de México, 22800, Ensenada, Baja California, México.
[3]Centro de Investigación Científica y de Educación Superior de Ensenada, Carretera Tijuana-Ensenada 3918, Apdo. Postal 22860 Ensenada, Baja California, Mexico

*Corresponding authors: H.A.B.-N. (hborbon@uabc.edu.mx), J. G.-S. (guerrero@ens.cnyn.unam.mx)

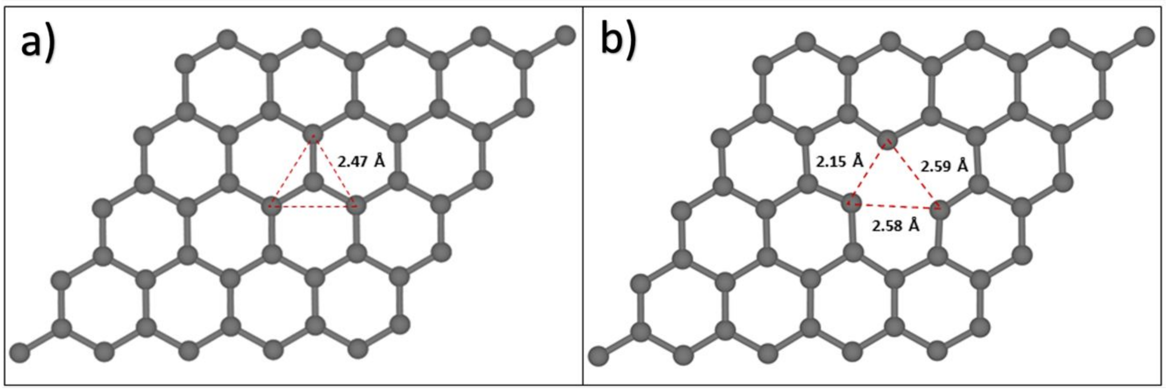

Figure S1. Top view of a) pristine graphene and b) graphene with a monovacancy.

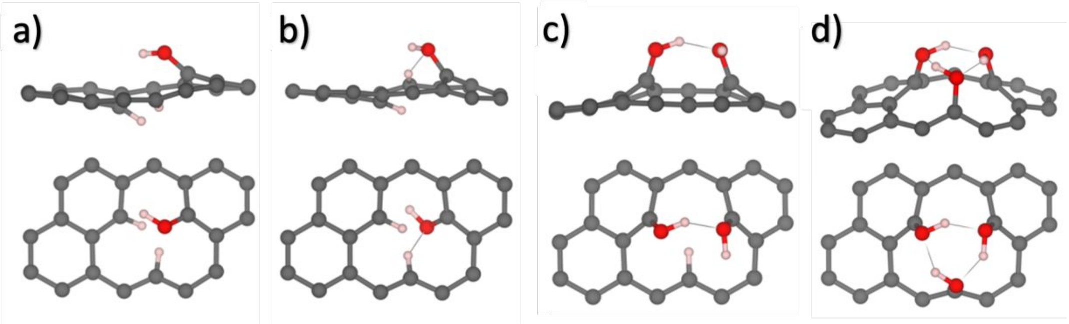

Figure S2. Top and side views of the less stable configurations for graphene with one hydroxyl group a) 1OH1, b) 1OH3, two hydroxyl groups c) 2OH1, and three hydroxyl groups d) 3OH1. Gray, white, and red spheres represent carbon, hydrogen, and oxygen atoms.

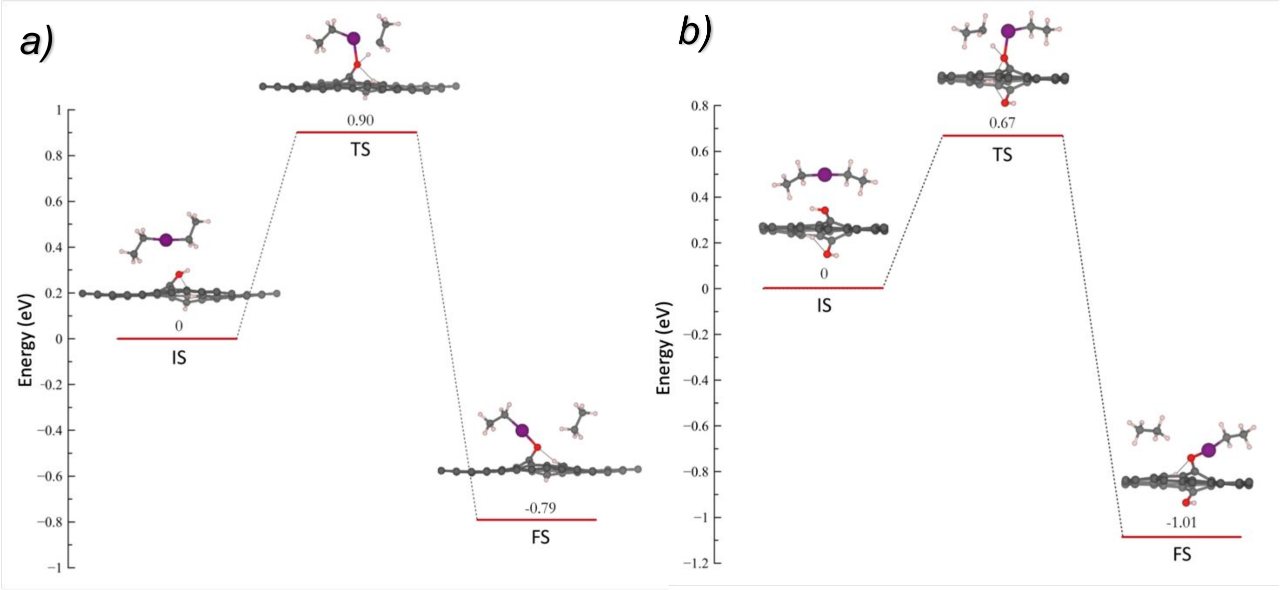

*Figure S3. Minimum energy pathway of the f*irst partial reaction of monovacancy graphene with a) one hydroxyl group and b) two hydroxyl groups.

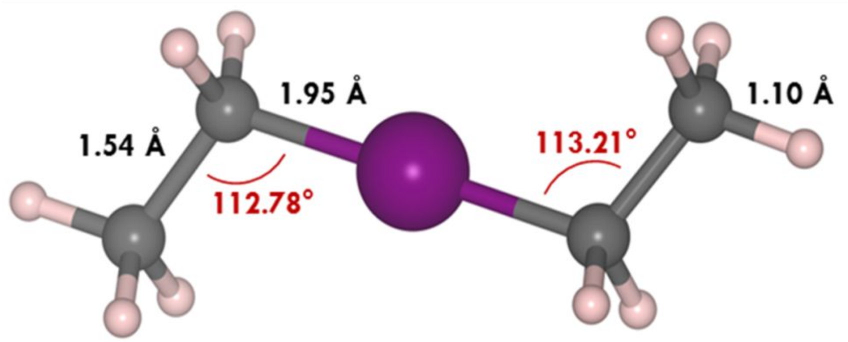

Figure S4. Side view of the relaxed DEZ molecule indicating the bond distance and bond angles.

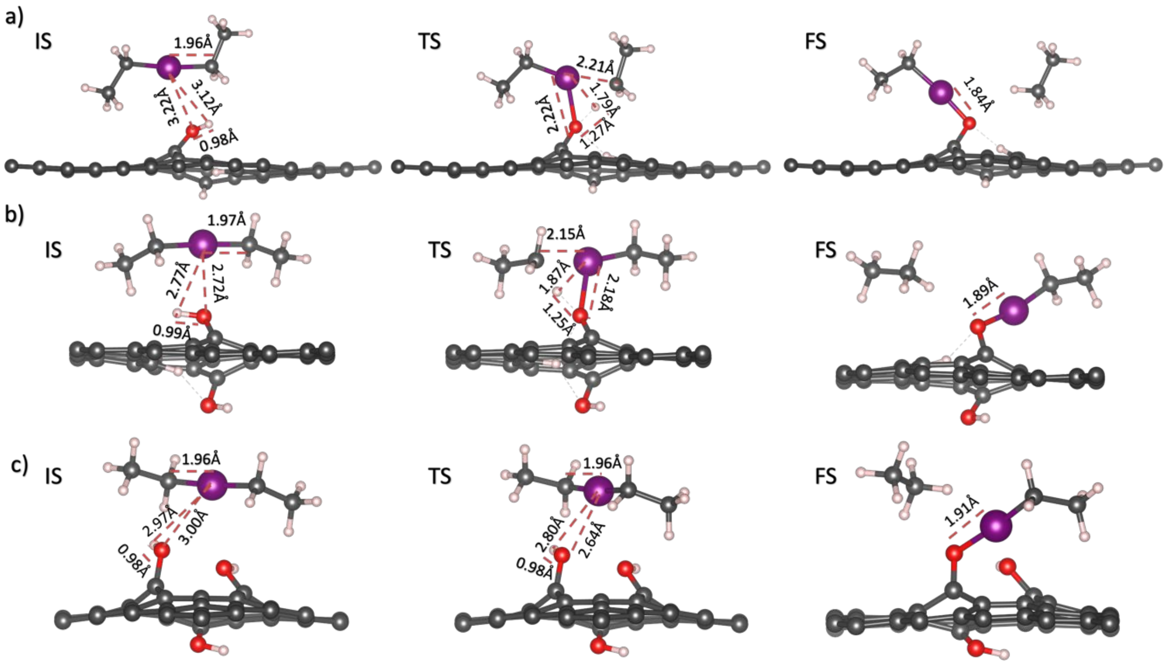

Figure S5. Side views and interaction distances between the main atoms in the partial reaction of DEZ for the initial, transition, and final states for the most stable structures a) 1OH, b) 2OH, and c) 3OH.

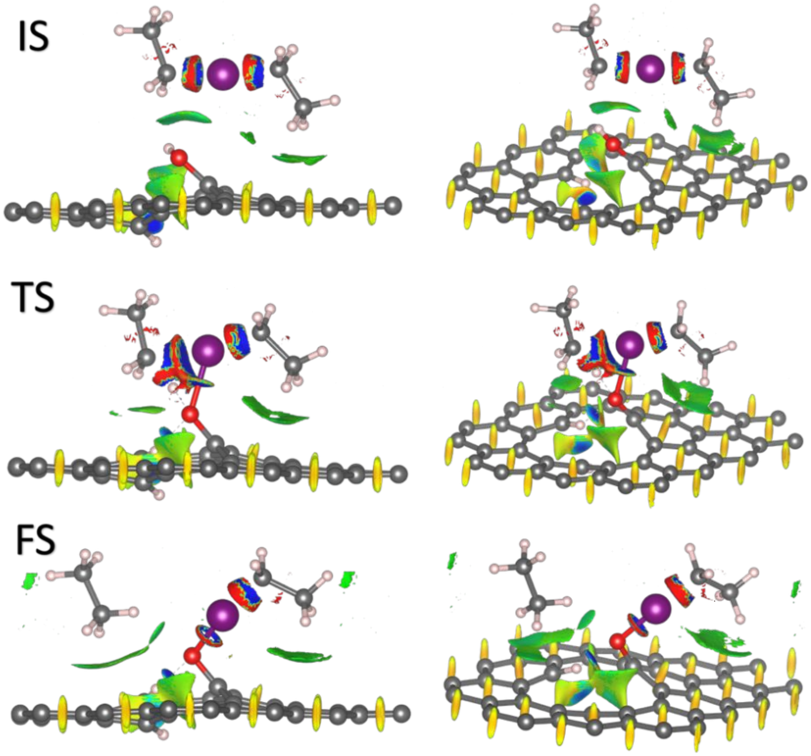

Figure S6. Top and side views of isosurface of the NCI of the 5x5 graphene monolayer with an asymmetric monovacancy interacting with one hydroxyl groups.

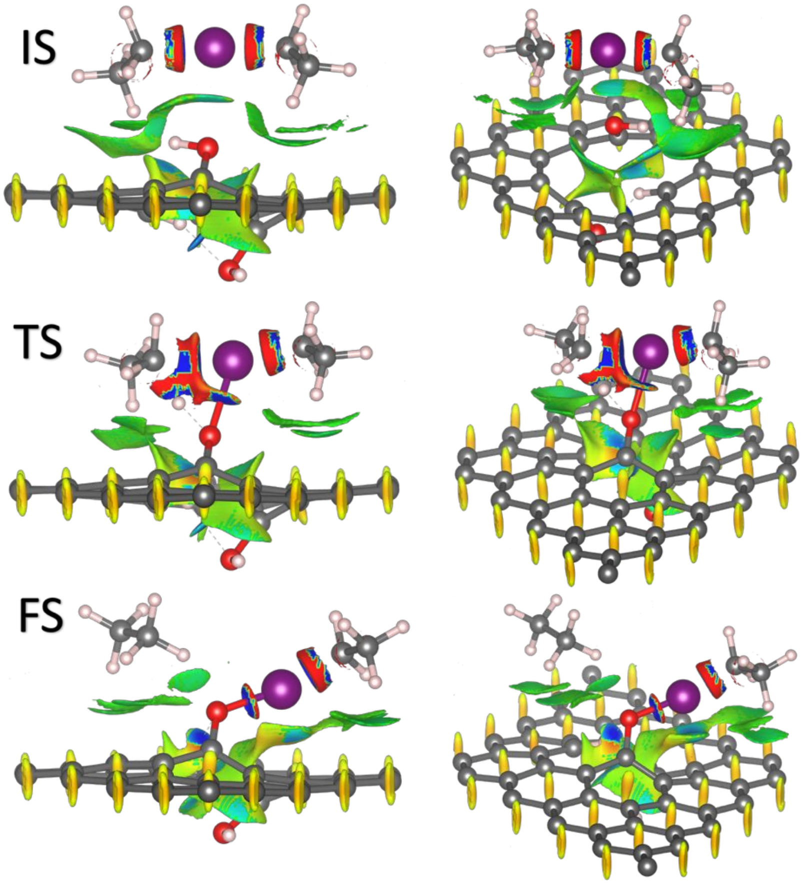

Figure S7. Top and side views of isosurface of the NCI of the 5x5 graphene monolayer with an asymmetric monovacancy interacting with two hydroxyl groups.

Non covalent interactions (NCI)

Non-covalent interactions (NCIs) are fundamental forces that dictate the structure, stability, and behavior of molecular systems. In contrast to covalent bonds, which involve the sharing or transfer of electrons, NCIs arise from weaker forces such as electrostatic attractions, dipole-dipole interactions, and dispersion forces. NCIs are pivotal in determining bonding types in materials and surface adsorption phenomena despite their relatively low strength compared to covalent bonds.

The non-covalent interaction (NCI) index was developed to analyze atomic and molecular interactions in periodic solids. This method enables the visualization and graphical representation of NCIs in three-dimensional space by analyzing electron density distributions and their associated reduced gradients.

The reduced density gradient (s) is a scalar field derived from the electron density ρ and is defined as:

$$s = \frac{1}{2(3\pi^2)^{1/3}} \frac{|\nabla\rho|}{\rho^{4/3}}.$$

When an inter- or intramolecular interaction occurs, a critical change appears in the reduced density gradient between the interacting atoms, generating critical points within the electron

density field. The reduced density gradient is a dimensionless quantity that describes deviations from a uniform electron distribution. The reduced gradient exhibits large positive values in regions distant from nuclei, where the electron density decays exponentially towards zero. In contrast, in regions associated with non-covalent interactions, the reduced gradient assumes small values, approaching zero.
Thus, to identify regions where non-covalent interactions occur, two simultaneous conditions must be satisfied:

(i) The reduced density gradient must be small (regions should be close to a critical point of the electron density).
(ii) The electron density must be low (no covalent bonds should exist in the region of interest).

Plotting isosurfaces of low *s* values in regions with low ρ values makes it possible to obtain a map of the density overlap, which corresponds to Pauli intermolecular repulsion. The graphical representation resulting from these reduced density gradient isosurfaces uses a color scale associated with the strength of the interaction. The strength is typically estimated through the product of the electron density and the second eigenvalue λ_2 of the Hessian matrix of the electron density at each point on the isosurface, where the sign of λ2 determines the attractive or repulsive character. A negative λ_2 suggests attractive interaction, such as hydrogen bonds or van der Waals forces, while positive λ_2 values indicate nonbonding interactions, repulsive interactions, or steric effects.
The 3D isosurfaces are defined by the product $\lambda_2\rho$, based on an RGB (Red-Green-Blue) scheme, where it is possible to define three kinds of interactions: non-bonding interactions or steric effects (red), weak interactions or van der Waals (vdw) interactions (green), and bonding interactions (blue). This method directly represents and characterizes non-covalent interactions in three-dimensional space [1-4].

[1] Contreras-García, J., Johnson, E. R., Keinan, S., Chaudret, R., Piquemal, J. P., Beratan, D. N., Yang, W. "NCIPLOT: A Program for Plotting Noncovalent Interaction Regions". J. Chem. Theory Comput. 7, 3, 625-632 (2011). DOI: https://doi.org/10.1021/ct100641a.

[2] Johnson, E. R., Keinan, S., Mori-Sánchez, P., Contreras-García, J., Cohen, A. J., Yang, W. "Reveling Noncovalent Interactions". J. Am. Chem. Soc. 132, 18, 6498-6506 (2010). DOI: https://doi.org/10.1021/ja100936w.

[3 Otero-de-la-Roza, A., Blanco, M. A., Pendás, A. M., Luaña, V. "Critic: a new program for the topological analysis of solid-state electron densities". Computer Physics Communications 180, 1, 157-166 (2009). DOI: https://doi.org/10.1016/j.cpc.2008.07.018.

[4] Otero-de-la-Roza, Johnson, E. R., Luaña, V. "Critic2: A program for real-space analysis of quantum chemical interactions in solids". Computer Physics Communications 185, 3, 1007-1018 (2014). DOI: https://doi.org/10.1016/j.cpc.2013.10.026.
